# A monolayer hiPSC culture system for autophagy/mitophagy studies in human dopaminergic neurons

**DOI:** 10.1080/15548627.2020.1739441

**Published:** 2020-04-14

**Authors:** Petros Stathakos, Natalia Jiménez-Moreno, Lucy A. Crompton, Paul A. Nistor, Jennifer L. Badger, Peter A Barbuti, Talitha L. Kerrigan, Andrew D. Randall, Maeve A. Caldwell, Jon D. Lane

**Affiliations:** a Cell Biology Laboratories, School of Biochemistry, University of Bristol, Bristol, UK; b Regenerative Medicine Laboratory, School of Clinical Sciences, University of Bristol, Bristol, UK; c Institute of Biomedical and Clinical Sciences, University of Exeter Medical School, Hatherly Laboratory, Exeter, UK; d Dementia Research Group, Institute of Clinical Neurosciences, School of Clinical Sciences, University of Bristol, Bristol, UK; e Trinity College Institute for Neuroscience, Trinity College Dublin, Dublin, Ireland

**Keywords:** Astrocyte, dopaminergic neuron, midbrain, Parkinson disease, stem cells

## Abstract

Macroautophagy/autophagy cytoplasmic quality control pathways are required during neural development and are critical for the maintenance of functional neuronal populations in the adult brain. Robust evidence now exists that declining neuronal autophagy pathways contribute to human neurodegenerative diseases, including Parkinson disease (PD). Reliable and relevant human neuronal model systems are therefore needed to understand the biology of disease-vulnerable neural populations, to decipher the underlying causes of neurodegenerative disease, and to develop assays to test therapeutic interventions *in vitro*. Human induced pluripotent stem cell (hiPSC) neural model systems can meet this demand: they provide a renewable source of material for differentiation into regional neuronal sub-types for functional assays; they can be expanded to provide a platform for screening, and they can potentially be optimized for transplantation/neurorestorative therapy. So far, however, hiPSC differentiation protocols for the generation of ventral midbrain dopaminergic neurons (mDANs) – the predominant neuronal sub-type afflicted in PD – have been somewhat restricted by poor efficiency and/or suitability for functional and/or imaging-based *in vitro* assays. Here, we describe a reliable, monolayer differentiation protocol for the rapid and reproducible production of high numbers of mDANs from hiPSC in a format that is amenable for autophagy/mitophagy research. We characterize these cells with respect to neuronal differentiation and macroautophagy capability and describe qualitative and quantitative assays for the study of autophagy and mitophagy in these important cells.

**Abbreviations:** AA: ascorbic acid; ATG: autophagy-related; BDNF: brain derived neurotrophic factor; CCCP: carbonyl cyanide m-chlorophenylhydrazone; dbcAMP: dibutyryl cAMP; DAN: dopaminergic neuron; DAPI: 4ʹ,6-diamidino-2-phenylindole; DAPT: N-[N-(3,5-difluorophenacetyl)-L-alanyl]-sphenylglycine; DLG4/PSD95: discs large MAGUK scaffold protein 4; DMEM: Dulbecco’s modified eagle’s medium; EB: embryoid body; ECAR: extracellular acidification rate; EGF: epidermal growth factor; FACS: fluorescence-activated cell sorting; FCCP: arbonyl cyanide p-triflouromethoxyphenylhydrazone; FGF: fibroblast growth factor; GAPDH: glyceraldehyde-3-phosphate dehydrogenase; GDNF: glia cell derived neurotrophic factor; hiPSC: human induced pluripotent stem cell; LAMP2A: lysosomal associated membrane protein 2A; LT-R: LysoTracker Red; MAP1LC3: microtubule associated protein 1 light chain 3; mDAN: midbrain dopaminergic neuron; MEF: mouse embryonic fibroblast; MT-GR: MitoTracker Green; MT-R: MitoTracker Red; NAS2: normal SNCA2; NEM: neuroprogenitor expansion media; NR4A2/NURR1: nuclear receptor subfamily group A member 2; OA: oligomycin and antimycin A; OCR: oxygen consumption rate; PD: Parkinson disease; SHH: sonic hedgehog signaling molecule; SNCA/α-synuclein: synuclein alpha; TH: tyrosine hydroxylase; VTN: vitronectin.

## Introduction

Parkinson disease (PD) is a progressive neurodegenerative disorder that causes motor, physical and cognitive impairments, leading to a reduction in quality of life and eventually, an inability to live independently. PD affects around 1% of the population aged over 65 and around 4% aged over 85. In the UK, there are currently ~145,500 PD patients, with the expectation that by 2065 the prevalence and incidence rates for PD in the UK will have almost doubled [1]. Classic PD symptoms (e.g., tremors at rest, postural instability, stiffness, dyskinesia) result from falling levels of the catecholaminergic neurotransmitter, dopamine, caused by the degeneration of midbrain dopamine-producing neurons (mDANs) [2]. Consequently, there is a need to better understand how PD pathophysiology affects these neurons. To achieve this goal, relevant and amenable cell-based assay systems are required for functional tests *in vitro*.

In PD, the most susceptible neurons are the neuromelanin-pigmented mDANs located in the *substantia nigra pars compacta* (SNpc; A9) [3]. To a lesser extent, dopaminergic neurodegeneration occurs in other brain regions such as the ventral tegmental area (A10) from where neurons project to the limbic system to establish the mesolimbic dopaminergic pathway. At later stages of the disease, neurodegeneration extends to other brain regions in a pattern that explains the later stage, non-motor symptoms of PD (e.g., depression, cognitive impairment, dementia) [4–6]. Although PD is primarily idiopathic, 5-10% cases are familial, with specific mutations associated with autosomal dominant and autosomal recessive forms [7]. To date, 21 genes with PD-causing mutations have been recognized, and several factors associated with increased risks of developing sporadic PD have been identified by meta-analysis [8] and genome-wide association studies/GWAS [9].

A prominent feature of PD is the formation of intracellular inclusions called Lewy Bodies, consisting mainly of filamentous SNCA (alpha-synuclein) protein aggregates that are toxic to mDANs [10]. Autophagy counters the accumulation of these toxic inclusions (see [11]), emphasizing the need for efficient cytoplasmic quality control. Furthermore, being extensively arborized, mDANs are especially sensitive to changes in energy homeostasis [12,13]. They employ L-type CACNA1D voltage-gated Ca^2+^ channels to provide tonic dopamine release at the striatum [12] via slow, broad action potentials [14,15]. This necessitates efficient energy-dependent [Ca^2+^] control processes – including transporter-mediated efflux across the plasma membrane [12,16–18] and mitochondrial Ca^2+^ sequestration [13] – and efficient mitochondrial quality control mechanisms. Tellingly, mitochondrial malfunctions have been strongly associated with PD (e.g. [19],), highlighting that efficient autophagy/mitophagy regulation is critical for mDAN functional maintenance.

The links between failing autophagy/mitophagy pathways and PD have been the focus of several studies on the causes of inherited PD. Autosomal dominant mutations in the retromer component, VPS35/PARK17, cause late-onset PD [20]. VPS35 facilitates retrograde transport of endosomal proteins to the *trans*-Golgi network or plasma membrane, and mutations have been suggested to interfere with LAMP2A-dependent chaperone-mediated autophagy (CMA) [21], ATG9A trafficking during macroautophagy [22], and DNM1 L/DRP1-mediated mitochondrial network dynamics [23]. Mitophagy can be triggered by PINK1-mediated PRKN recruitment to depolarized mitochondria via phosphorylation and ubiquitylation signaling, and recruitment of autophagy receptors [24–29]. Interestingly, PINK1 and PRKN are not essential for housekeeping mitophagy in flies and in mice [30,31], so further work is needed to understand the precise regulatory pathways controlling autophagy/mitophagy in mDANs.

Protocols for the generation of human mDANs *in vitro* for PD-related research include dopaminergic neural differentiation from embryonic stem cells (ESCs) or human induced pluripotent stem cells (hiPSCs), and direct conversion of stem cells or fibroblasts by forced expression of dopaminergic neuronal factors (see [32]). hiPSC technology provides a flexible and relatively straightforward approach to study the underlying causes of neural disturbances *in vitro*, while also being amenable for cellular transplantation [33–36]. Differentiated cells (e.g., skin fibroblasts) are re-programmed toward a stem cell state through the addition of factors first described by Yamanaka [37]. hiPSC lines can then be differentiated into mDANs through the timely addition of a cocktail of growth and patterning factors, and cytokines and inhibitors that mimic *in vivo* dopaminergic neural developmental process. To specify midbrain mDANs, these include: SMAD signaling inhibitors to suppress mesodermal fate (e.g., LDN193189, NOG [noggin], SB431542); patterning morphogens (e.g., FGF8, SHH, WNT); mitogens (e.g., EGF, heparin); and survival/dopaminergic identity factors (e.g., BDNF, GDNF) [32]. Non-adherent/embryoid-body (EB) or adherent monolayer differentiation protocols can be employed, each having particular advantages and disadvantages. By whichever approach, mDANs need to: (i) co-express neuronal (e.g., TUBB3/βIII-tubulin/TUJ1), dopaminergic (e.g., TH [tyrosine hydroxylase]), and midbrain (e.g., FOXA2, LMX1A) markers; (ii) be able to survive *in vitro*, and elongate axons with evidence of synapses; (iii) be able to synthesize dopamine; (iv) display evidence of electrical activity; (v) have the capability to reverse PD-like symptoms in animal models without side effects if to be used for cell-based therapies [38].

Despite the obvious potential of hiPSCs for studying autophagy pathways in human mDANs, relatively few PD-related studies have been attempted to date using this approach, and most did not provide a full characterization of the cells under study (for discussion, see [39]). This argues for the need for efficient, reliable hiPSC differentiation protocols to obtain sufficient numbers of confirmed mDANs for single-cell and population-based autophagy/mitophagy assessments in PD. Here, we describe an improved, monolayer protocol that can reliably and rapidly generate >70% verified human mDANs in a format that enables imaging-based autophagy studies in PD-relevant cells. We present a basic characterization of the biology of these cells, including temporal transcriptional profiling of several macroautophagy/mitophagy genes. We suggest that this protocol will be useful for labs wishing to study the basic mechanisms of autophagy regulation in human mDANs *in vitro*, how disease-associated mutations alter these parameters, and for use as an amenable drug-screening platform for PD research.

## Results and discussion

### Comparing differentiation protocols for hiPSC-derived mDANs

We compared several existing hiPSC differentiation protocols for human mDANs. Firstly, an EB-based protocol that was originally configured to generate basal forebrain cholinergic neurons [40] was adapted to promote mDAN differentiation. We omitted EGF (favors glial differentiation [41,42]), and included: purmorphamine (SHH agonist); FGF8/FGF8A; ascorbic acid (AA; improves mDAN survival [43]); BDNF (improves neuronal survival through activation of NTRK2 receptors, and enhances dopamine release/uptake [44,45]); GDNF (promotes mDAN growth and survival [46]); db-cAMP (upregulates TH synthesis, and promotes neuronal differentiation/survival [47–49]); DAPT (NOTCH inhibitor that promotes neuronal differentiation [50]); and TGFB3 (increases responsiveness to GDNF, and promotes neurite outgrowth and neuronal survival [51]; Figure S1A). Cell populations generated labeled strongly for TUBB3, TH, and FOXA2, indicating the presence of abundant mDANs (Figure S1B and C). Cell bodies were mostly located within the EB, with abundant axons extending radially for several mm (Figure S1B and C), meaning that cell characterization could only be accurately carried out on cells migrating beyond the periphery of the EB. Focusing on those cells at differentiation day 55–60, 92.0 ± 0.9% were TUBB3-positive, of which 28.3 ± 0.7% were also TH-positive (i.e., 26.3 ± 0.7% of total cells counted; means ± SEM [Figure S1D]). Moreover, FOXA2 was expressed in 75.6 ± 1.4% of these cells, and 29.2 ± 0.9% of these were also TH-positive (22.3 ± 0.9% of total cells; Figure S1E). Longer differentiation times (75–80; 95–100 d) did not increase the numbers of FOXA2-positive midbrain cells; however, the proportion of measurable TH-positive cells did increase significantly, with mDAN numbers peaking at ~55% (Figure S1 F).

Despite the good yield of mDANs generated using this EB-based protocol (Figure S1), it soon became clear that autophagy assessments would be very challenging. Achieving high numbers of mDANs required prolonged total time in culture (>100 d), which is restrictive and costly while isolating individual cells for imaging studies of cell bodies and axons, or flow cytometry analysis, was not straightforward. Poor antibody and/or viral penetrance into the EB meant that accurate characterization and functional studies would also be limited (data not shown); meanwhile, neurons generated using this protocol tended to cluster together and peel away from the coverslip surface upon gentle agitation (data not shown). To arrive at a more reliable mDAN culture method, we therefore compared the practical ease and effectiveness of other published differentiation methods optimized for neurons in monolayers (namely, the Jaeger et al. [52] and Kirkby et al. [53] protocols), against our own mDAN-directed modification of the neocortical differentiation method described by Nistor et al. [54,55] – itself a xeno- and feeder-free adaption of the Shi et al. protocol [54,55].

In pilot neuralizations, the yield of midbrain FOXA2-positive cells was high using the Jaeger et al. method [52] (65.6 ± 3%), but the proportion of cells expressing TH did not exceed 12% of the total cell population (11.7 ± 0.9%; data not shown). Meanwhile, in our hands, the Kirkeby et al. protocol [53] generated lower mDAN numbers in a single neuralization attempt (data not shown). Clearly, further optimization would have improved mDAN efficiency using these protocols; however, based on the outcome of pilot neuralizations (see below), we focused on adapting the Nistor et al. protocol for the specification of mDANs for our studies. To do so, we supplemented N2B27 differentiation media with the neural fate inducing factors LDN193189 (100 nM) and SB431542 (10 μM), and the regional specification factors, SHH-C24II (200 ng/ml) and CHIR99021 (a GSK3 inhibitor/WNT signaling activator; 0.8 μM) (Figure S2A). Using this approach, we generated healthy, dispersed neuronal cultures, with relatively high numbers of FOXA2/TH-positive neurons (~60% TUBB3-positive; ~43% FOXA2-positive; ~11% TH/FOXA2-positive: Figure S2B and C).

### An optimized protocol for the rapid and efficient generation of human mDANs from hiPSCs

**Figure 1.**
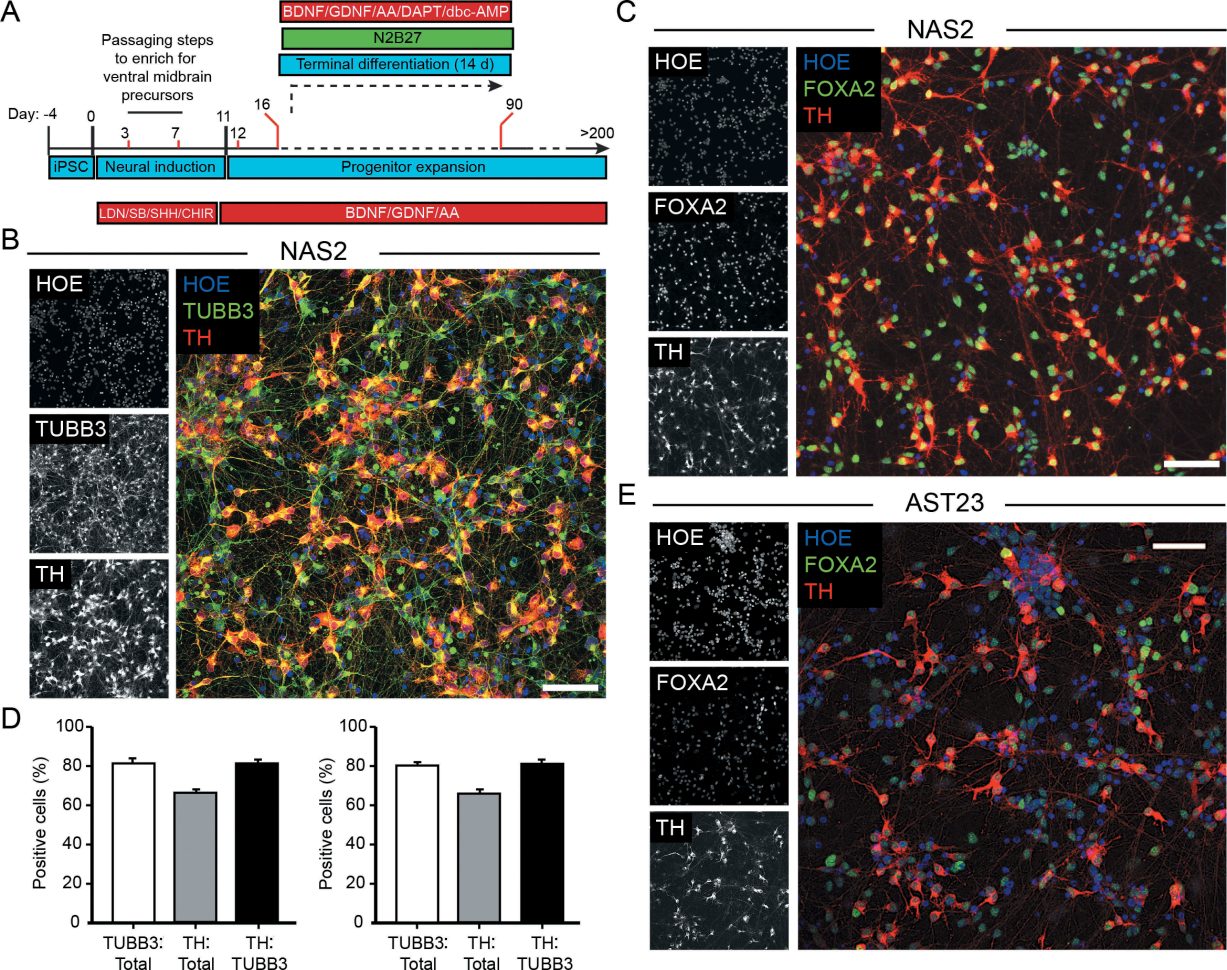
The final optimized monolayer hiPSC mDAN differentiation protocol. (A) Schematic of the protocol. Refer to Materials and Methods for concentrations. Passaging steps are indicated with red lines. Detailed, stepwise methodology can be found in [80]. (B, C) Confocal immunofluorescence imaging of day 35 neuronal cultures (NAS2 hiPSCs) generated using the final protocol. (B) Cultures labeled with anti-TUBB3 (TUJ1) and anti-TH antibodies and stained with Hoescht. (C) Cultures labeled with anti-FOXA2 and anti-TH antibodies and stained with Hoescht. (D) Quantitation of cell identity in day 35 neuronal cultures. Data show means ± SD for 3 independent terminal differentiations seeded from a single neuralization. Four fields per coverslip were analyzed. (E) Confocal immunofluorescence imaging of day 35 AST23 cultures. Bars: 50 μm

Our final hiPSC differentiation protocol is shown schematically in Figure 1A, with example fields of NAS2-derived cells co-labeled for TH and either TUBB3 or FOXA2 shown in Figure 1B, C. Using this protocol with the NAS2 hiPSC line, we routinely achieved TH-positive cell yields (TH/total) exceeding 65% (67.1 ± 1.3% at differentiation day 30–35; means ± SD; Figure 1D), within a neuronal population of 82.1 ± 1.3% (TUBB3-positive/total cells), of which 81.8 ± 1.3% neurons were also TH-positive (i.e., TH- and TUBB3-positive; Figure 1D). Importantly, the midbrain identity of these cells was very high (81.1 ± 1.1% FOXA2-positive/total cells), with 81.9 ± 1.2% of these co-expressing TH (i.e., TH- and FOXA2-positive mDANs; Figure 1D). We have successfully repeated this protocol using other iPSC and ES cell lines, including AST23 (see Figure 1E), SHEF6, MSU, and PAR3B (data not shown).

**Figure 2.**
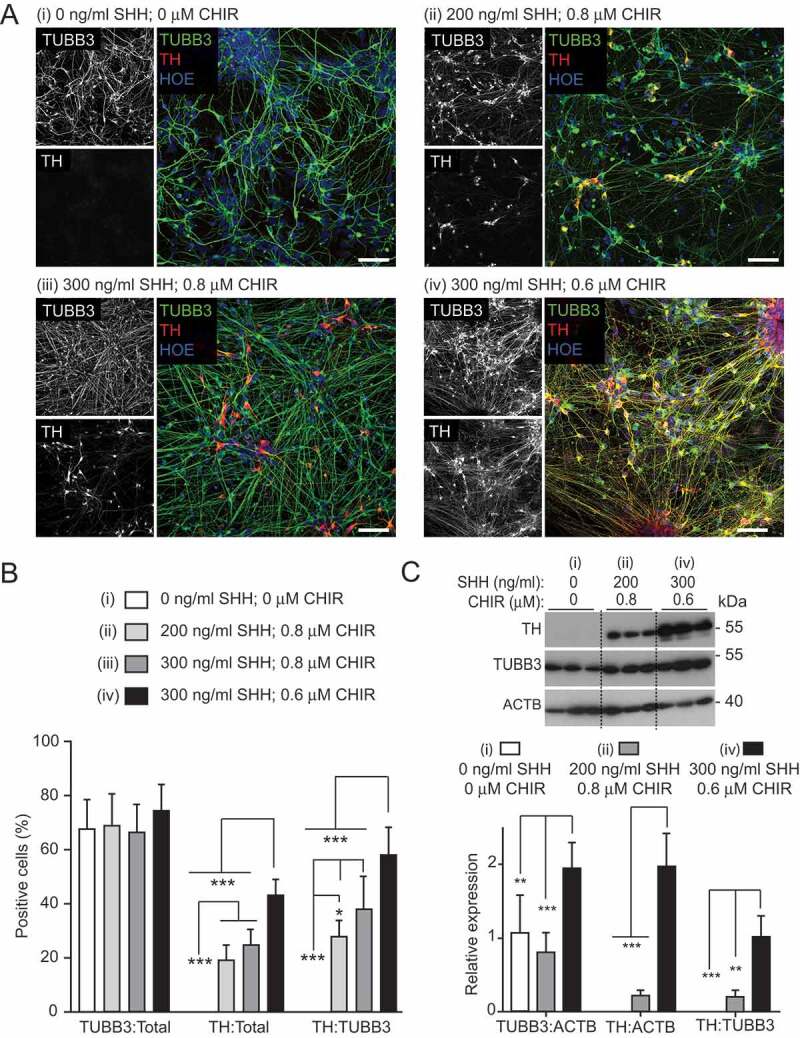
Optimizing SHH-C42II and CHIR99021 concentrations during neural induction for improved mDAN differentiation efficiency. Images (A) and quantification (B) of day 35 mDAN cultures (NAS2 hiPSCs) labeled with anti-TUBB3 (TUJ1) and anti-TH antibodies and counter-stained with Hoescht. 4 separate conditions were tested: (I) no SHH, no CHIR99021; (ii) 200 ng/ml SHH, 0.8 μM CHIR99021; (iii) 300 ng/ml SHH, 0.8 μM CHIR99021; (iv) 300 ng/ml SHH, 0.6 μM CHIR99021. Data show means ± SD for 3 independent terminal differentiations seeded from a single neuralization. Four fields per coverslip were analyzed. (C) Immunoblotting for TH expression in lysates of cells at differentiation day 30, generated using conditions (i), (ii) and (iv). * P < 0.05; ** P < 0.01; *** P < 0.001. Bars: 50 μm

To arrive at this efficient protocol, we first confirmed that inclusion of FGF8 did not improve the proportion of midbrain cells (49.5 ± 10% FOXA2-positive cells without FGF8; 44.8 ± 10.8% with FGF8; Figure S3A and B) [53,56], whereas increasing GDNF and DAPT concentrations (to 20 ng/ml and 10 μM, respectively) resulted in an almost 2-fold increase in TH-positive DANs (11.1 ± 5.7% at lower versus 19.8 ± 5.4% at higher concentrations; total TUBB3-positive neuronal populations were comparable, 62.7 ± 8.0% at lower versus 69.1 ± 11.5% at higher concentrations) (Figure S3 C and D). We next postulated that increasing the concentration of SHH-C24II, while simultaneously decreasing the concentration of the WNT agonist, CHIR99021, would enrich for mDANs. This is because dorsoventral specification is mediated by gradient competition between these factors [57]. Four combinations were tested in parallel neuralizations (NAS2 hiPSC line): (i) no SHH-C24II, no CHIR99021; (ii) 200 ng/ml SHH-C24II, 0.8 μM CHIR99021 (as used in previous studies [53,56]); (iii) 300 ng/ml SHH-C24II, 0.8 μM CHIR99021; (iv) 300 ng/ml SHH-C24II, 0.6 μM CHIR99021 (Figure 2A,B). Although the total neuronal population remained similar in all four combinations (means ± SD: [i] 68 ± 10.3%; [ii] 69.1 ± 11.5%; [iii] 67 ± 9.5%; [iv] 74.6 ± 9.2%), the DAN percentage of both the total cell (TH-positive/total cells: [i] no TH-positive cells; [ii] 19.8 ± 5.4%; [iii] 25.2 ± 5.3%; [iv] 43.3 ± 5.8%) and the neuronal population (TH/TUBB3-positive cells: [i] no TH-positive cells; [ii] 28.6 ± 5.6%; [iii] 38.5 ± 11.55%; [iv] 58.7 ± 9.5%) were significantly higher using 300 ng/ml SHH, 0.6 μM CHIR99021 (Figure 2B). Using this optimal condition, almost all the TH-positive cells co-expressed FOXA2 (~ 99%), suggesting that the neurons generated were of midbrain, dopaminergic specification (i.e., mDANs). Immunoblotting for TH at differentiation day 25–30 supported the imaging data, in that at the optimal SHH-C24II/CHIR99021 concentrations, TH expression was highest (Figure 2C).

We hypothesized that improving the early production of midbrain-identity cells in the culture would be an effective way to increase the final yield of mDANs. To achieve this, we introduced additional passaging steps at differentiation days 3 and 7 of neural induction and patterning (see Figure 1A), based on reports demonstrating that expression of the pluripotency marker, POU5F1/OCT4, is almost completely diminished at day 3 of neural induction/patterning [52,53,56,58]. To select for established ventral mDAN progenitors, we dissociated (with Accutase) and passaged cells on poly-ornithine/laminin-coated dishes, as laminin favors neuronal progenitor attachment [59]. As shown in Figure S4, this yielded neural progenitor populations that were almost entirely FOXA2- and LMX1A-positive (differentiation day 12) – thereby confirming floor plate mDAN progenitor status.

### Characterization of mDAN cultures generated using the optimized monolayer protocol

**Figure 3.**
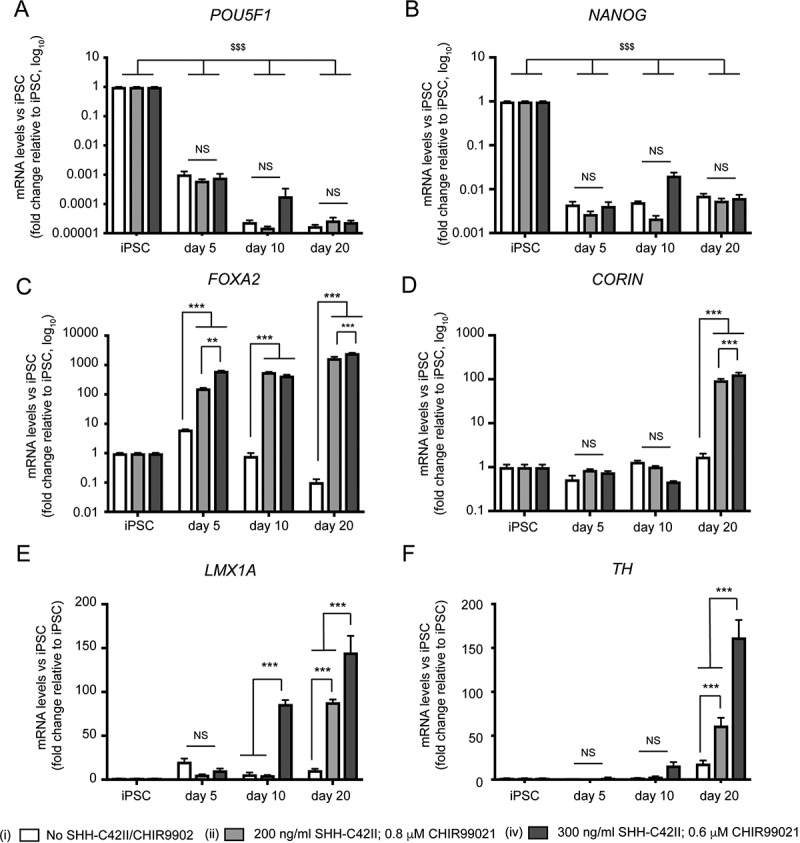
qRT-PCR expression profiling of pluripotency, midbrain, and dopaminergic markers during early differentiation under different SHH-C42II/CHIR99021 combinations in neuronal cultures enriched for mDANs. Differentiation was carried out using the optimized monolayer protocol, using NAS2 hiPSCs, with SHH-C42II/CHIR99021 ratios as follows: (I) no SHH-C42II, no CHIR99021; (ii) 200 ng/ml SHH-C42II, 0.8 μM CHIR99021; (iv) 300 ng/ml SHH-C42II, 0.6 μM CHIR99021. Results are from a single neuralization, and values are presented as means ± SEM of 3 terminal differentiations, fold-change versus hiPSCs. (A, B) Data are compared against hiPSC levels for these pluripotency markers, where: $$$ P < 0.001. (C-F) For these midbrain, floorplate and dopaminergic markers, data are compared within time-points and within treatments. Statistical comparisons against hiPSCs are not shown for clarity. ** P < 0.01; *** P < 0.001

To further characterize mDAN cultures generated using the optimized, monolayer protocol, we carried out qRT-PCR of a range of relevant pluripotency and ventral midbrain marker genes in our neuronal cultures enriched for mDANs. We compared conditions: (i) no SHH-C24II, no CHIR99021; (ii) 200 ng/ml SHH-C24II, 0.8 μM CHIR99021; (iv) 300 ng/ml SHH-C24II, 0.6 μM CHIR99021 (Figure 3 shows data from a single neuralization representative of 3 repeats with similar outcomes using the NAS2 hiPSC line). Expression of pluripotency genes POU5F1 and NANOG declined by day 5 in all conditions, and remained low thereafter, demonstrating that the hiPSCs had undergone differentiation (Figure 3A,B). FOXA2 expression was significantly greater at day 5 in the 2 SHH/CHIR combinations tested compared to the no SHH-C24II/CHIR99021 control and remained elevated thereafter demonstrating induction of midbrain transcripts (Figure 3C). Expression of CORIN (a floorplate marker) was initiated later (day 20), but once again, only in the SHH-C24II/CHIR99021 treated cultures where it was significantly increased compared to the control (Figure 3D). Expression of the midbrain LIM family transcription factor LMX1A increased significantly at day 10 only in the 300 ng/ml SHH-C24II/0.6 μM CHIR99021 group (iv) but needed 20 d to significantly increase with the 200 ng/ml SHH-C24II/0.8 μM CHIR99021 combination (ii) (Figure 3E). Even at this stage, we recorded a significant difference in LMX1A expression between the two SHH-C24II/CHIR99021 combinations (Figure 3E). Importantly, expression of TH was found to increase significantly at both SHH-C24II/CHIR99021 combinations at day 20, but the 300 ng/ml SHH-C24II/0.6 μM CHIR99021 combination gave the strongest TH induction (Figure 3F). Together, these data are consistent with the immunological characterization suggesting that combination (iv) 300 ng/ml SHH-C24II/0.6 μM CHIR99021 was best suited for mDAN patterning and that 20 d of differentiation is the minimum required to trigger expression of important midbrain, dopaminergic markers.

**Figure 4.**
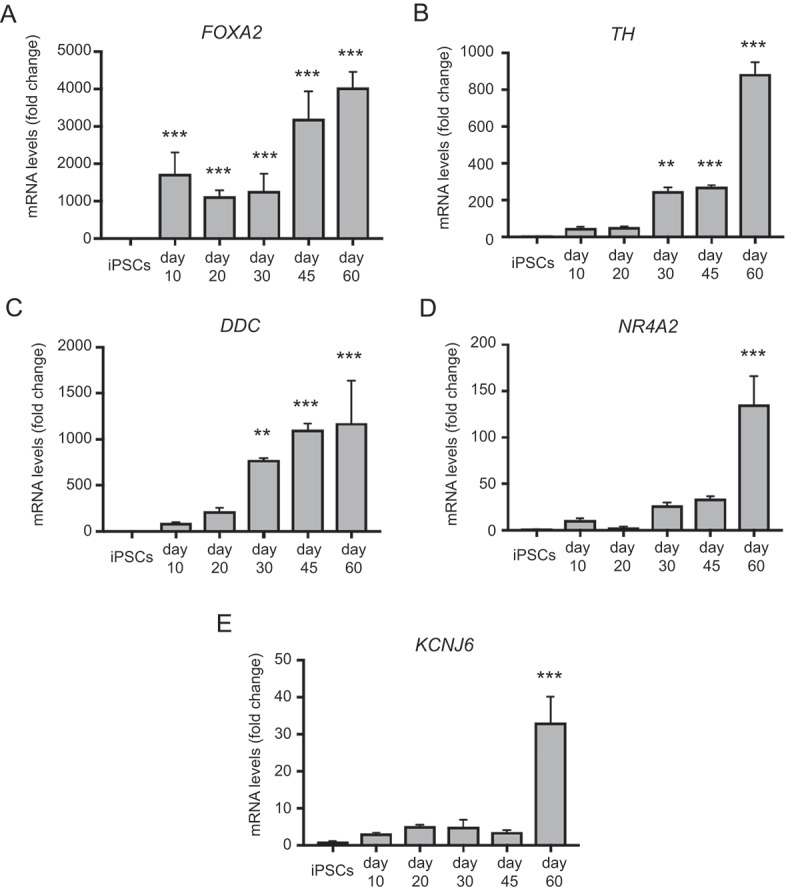
qRT-PCR analysis of temporal changes in midbrain/floorplate and A9 dopaminergic maturity markers in neuronal cultures enriched for mDANs. NAS2 hiPSCs were differentiated according to the optimized monolayer protocol, using 300 ng/ml SHH-C42II, 0.6 μM CHIR99021. (A) Floorplate/midbrain marker FOXA2. (B-D) Dopaminergic markers, TH, DDC, and NR4A2. (E) The A9 dopaminergic maturity marker, KCNJ6. Results are from a single neuralization, and values are presented as means ± SEM of 3 terminal differentiations as fold-change from hiPSCs. ** P < 0.01; *** P < 0.001

To temporally define midbrain dopaminergic gene expression, we carried out a qRT-PCR analysis of our mDAN-enriched neuronal cultures differentiated for up to 60 d in the optimal SHH-C24II/CHIR99021 combination; i.e., condition (iv) 300 ng/ml SHH-C24II/0.6 μM CHIR99021. Changes in *FOXA2* expression appeared biphasic, increasing early on day 10, then again at day 45 (Figure 4A), whereas expression of dopaminergic markers *TH* and *DDC* (DOPA decarboxylase) increased significantly only by day 30 (Figure 4B,C). *TH* showed a further sharp upturn between days 45–60 suggesting that prolonged differentiation favors dopaminergic identity (Figure 4B). Expression of *NR4A2*/*NURR1* – a nuclear transporter that supports dopaminergic status [60,61] – and *KCNJ6* – a potassium channel needed to support dopaminergic neuronal function – remained relatively low until day 60 (Figure 4D,E). Together, these data clarify the temporal nature of key gene expression in our monolayer differentiation system: using this protocol, it is possible to generate abundant FOXA2- and TH-positive mDANs within 25–30 d for imaging-related studies, but a longer differentiation period of 45–60 d is advised for studies in which maximal expression of many key midbrain, floorplate and/or dopaminergic markers is necessary (e.g., for physiological experiments). Interestingly, maturing mDAN cultures contained abundant astrocytes (as determined by anti-S100B immunostaining; Figure S5A), indicating that glial support is present in these cultures, and relative glial numbers increased with differentiation phase duration (from ~10% at day 30 to ~25% at day 50–60; Figure S5B). mDANs at differentiation day 50 expressed the synaptic marker DLG4 (PSD95) in punctate structures (Figure S6A); meanwhile, electrophysiological analysis showed that the differentiated neurons could fire action potentials and have voltage-gated K^+^ and Na^+^ currents at day 70 (15 out of 20 patched cells shared this profile; Figure S6B-D).

### Preliminary assessment of macroautophagy capability in hiPSC-derived mDAN cultures

**Figure 5.**
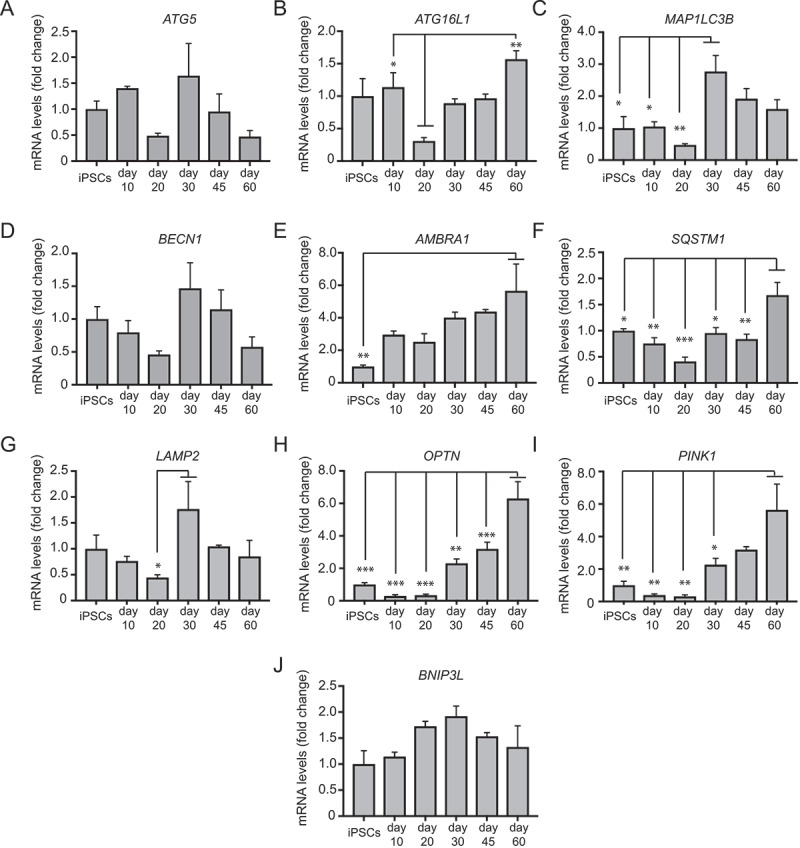
qRT-PCR analysis of the temporal expression of autophagy genes as shown during mDAN differentiation in neuronal cultures enriched for mDANs. NAS2 hiPSCs were differentiated according to the optimized monolayer protocol, using 300 ng/ml SHH-C42II, 0.6 μM CHIR. Results are from a single neuralization, and values are presented as means ± SEM of 3 terminal differentiations as fold-change from hiPSCs. * P < 0.05; ** P < 0.01; *** P < 0.001

To test the suitability of our hiPSC-derived mDANs for autophagy research, we first used qRT-PCR to assess the timings of expression of key macroautophagy and mitophagy genes, from the hiPSC stage to day 60 of differentiation (Figure 5). Overall, changes in expression were relatively small, but some interesting patterns emerged. *ATG5, MAP1LC3B, BECN1/Beclin 1*, and *LAMP1* showed similar profiles, with an initial drop in expression at around day 20 relative to the hiPSC stage, and expression rising again at day 30 to decline once more by day 60 (Figure 5A,C,D,G; significant only for *MAP1LC3B* and *LAMP2*). *ATG16L1* and the autophagy receptors *SQSTM1* and *OPTN* showed an initial decline in expression at day 20, followed by a gradual increase to day 60 (Figure 5B,F,H). *PINK1* followed a similar pattern (Figure 5I), but *BNIP3 L* (*NIX*) differed with expression changing little during differentiation (Figure 5J). Finally, *AMBRA1* expression showed a steady increase up to day 60 (Figure 5E). Broadly, these data are consistent with an initial decline in macroautophagy potential during early differentiation (day 10–20), followed by a regaining of macroautophagy gene expression as mDANs mature. Notably, re-activated expression of several macroautophagy genes (at day 30) correlated with expression of the dopamine synthesis enzymes, *TH* and *DDC* (Figure 4B,C).

**Figure 6.**
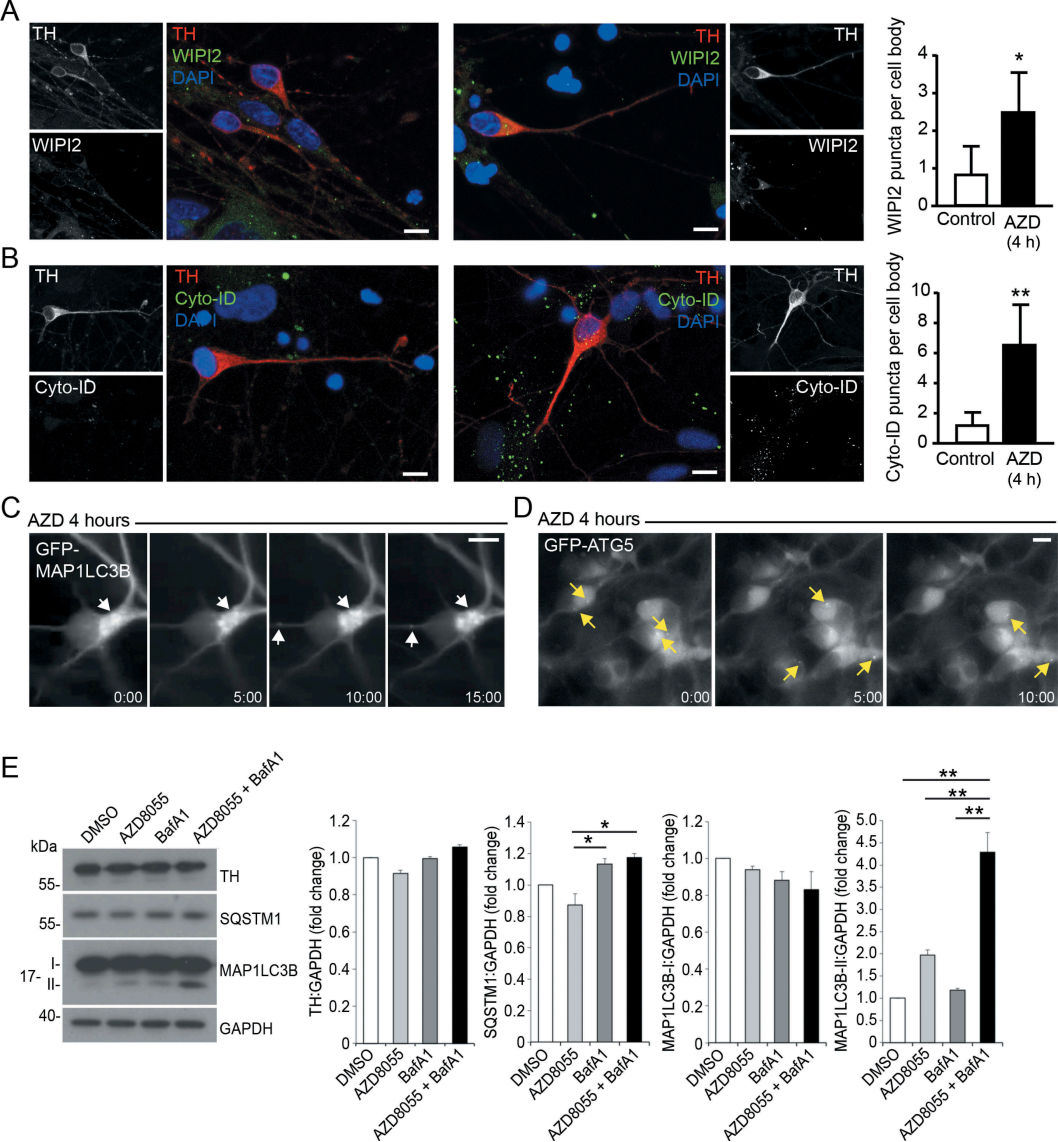
Examples of autophagy assessments in hiPSC-derived mDANs. In all cases, mDANs were differentiated from NAS2 hiPSCs using the optimized monolayer protocol. (A, B) Confocal imaging of day 60 neurons treated for 4 h with AZD8055, then stained for TH alongside (A) WIPI2 and (B) Cyto-ID. Example images to the left; quantitation to the right. (C) Day 47 neurons transduced with GFP-MAP1LC3B lentivirus and imaged up by time-lapse wide-field microscopy following the addition of AZD8055 (AZD; 4 h). Dynamic, GFP-MAP1LC3B-positive autophagic puncta can be observed within soma and neurites (arrows). (D) Day 25 neurons were transduced with lentivirus expressing GFP-ATG5, and imaged by time-lapse wide-field microscopy following the addition of AZD8055 (AZD; 4 h). GFP-ATG5 puncta (arrows) can be observed appearing, becoming brighter, then fading within the soma. (E) Representative immunoblots (left) for the indicated proteins from mDANs (differentiated at day 28–40) after 4 h treatment with AZD (1 μM) and/or BafA1 (20 nM), and densitometry quantitation (right). Arrow indicates LC3-II. Data are mean ± SEM (n = 2). Statistical analysis was performed using one-way ANOVA followed by a Tukey’s multiple comparisons test; P < 0.05; ** P < 0.01. Bars: 10 μm

**Figure 7.**
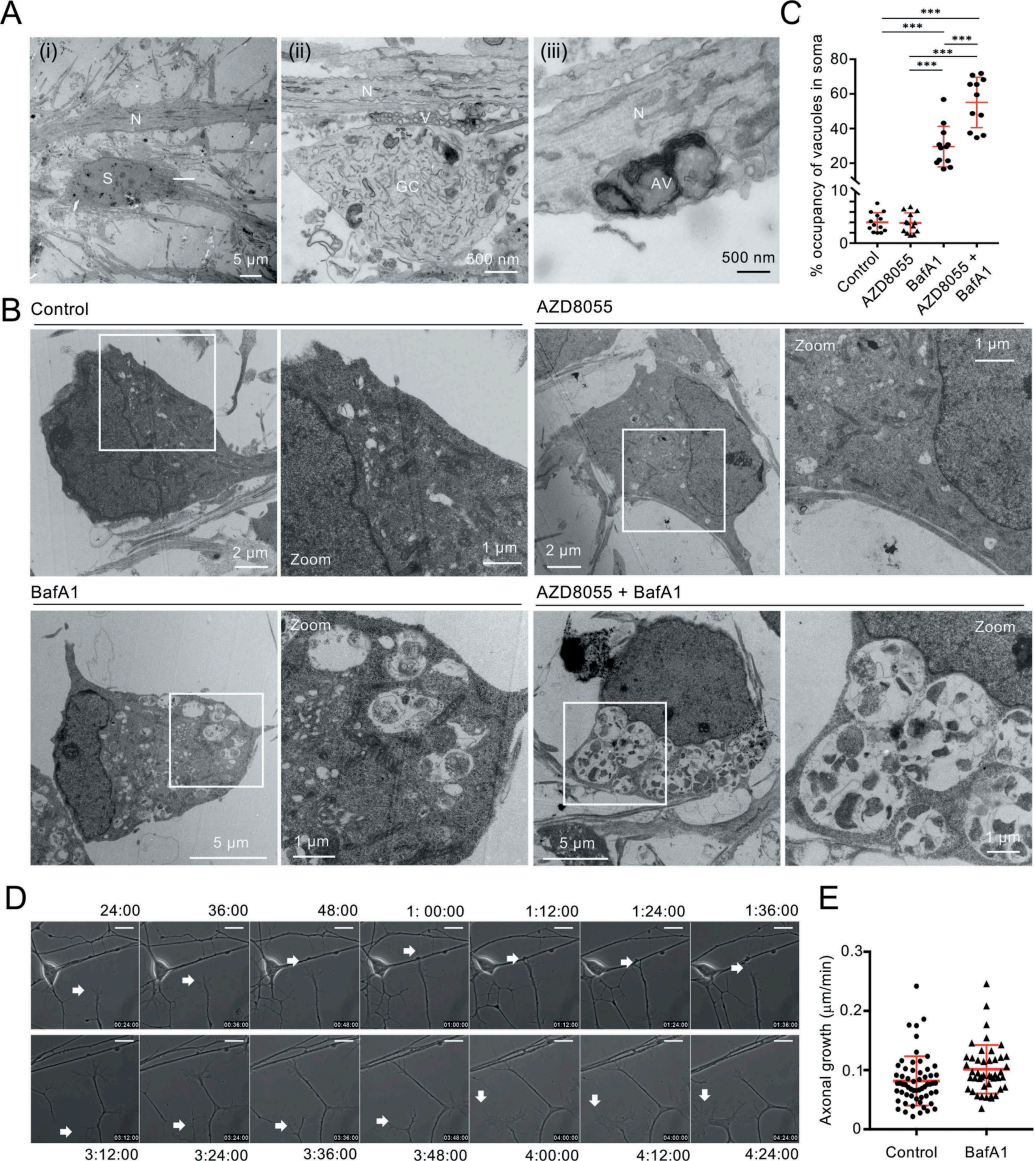
mDAN ultrastructure and growth cone dynamics. (A) Example EM images of untreated mDAN cultures: (I) shows a cell body (soma) region and neurite; (ii) shows a growth cone adjacent to neurites, with evidence of docked vesicular clusters; (iii) shows autophagosomal structures in a neurite. S = soma; N = neurite; GC = growth cone; AV = autophagic vacuole; V = vesicular cluster. (B) Example images and (C) quantitation (% cytoplasmic area occupancy) of autolysosomal profiles in the cell bodies of neurons present in mDAN cultures treated for 4 h with AZD8055 in the absence or presence of BafA1 (n = 12 cell bodies per condition). (D) Example frames from a live-cell imaging time-lapse sequence and (E) quantitation of axonal growth code extension rates in control or BafA1-treated mDAN cultures. Bar: 10 μm

To provide proof-of-principle macroautophagy analysis in hiPSC-derived mDANs, we tested: (i) immunostaining using anti-WIPI2 (Figure 6A) and anti-MAP1LC3B (data not shown) antibodies; (ii) CYTO-ID staining (Figure 6B); (iii) lentiviral GFP-MAP1LC3B expression (Figure 6C); (iv) lentiviral GFP-ATG5 expression (Figure 6D); (v) immunoblotting for MAP1LC3B lipidation and SQSTM1 turnover (Figure 6E); (vi) ultrastructural analysis of autolysosomal vacuoles (Figure 7A-C). Anti-WIPI2 antibodies worked well in methanol-fixed hiPSC-derived mDANs (Figure 6A), and WIPI2 puncta were found to significantly increase in TH-positive neurons in 60-d mDAN cultures treated with 1 μM AZD8055 (MTOR inhibitor; 4 h) compared with control, indicating enhanced autophagosome assembly site initiation (Figure 6A). On the other hand, the anti-MAP1LC3B antibodies that we have so far tested have not been successful due to difficulties distinguishing true autophagic puncta from fluorescent cell debris (formaldehyde and methanol fixation gave similar results; data not shown). Instead, we recommend the CYTO-ID kit for rapid autophagosome/autolysosomal staining in hiPSC-derived neurons (Figure 6B). This green fluorescent cationic amphiphilic tracer dye gives excellent overlap with MAP1LC3B-labeled puncta in RPE1 cells in our hands (data not shown), and enables vital staining of autophagic structures [62]. Importantly, it can be fixed, thus enabling co-staining with e.g., anti-TH antibodies (Figure 6B). AZD8055-treated mDANs were loaded with CYTO-ID, fixed with formaldehyde, and stained with anti-TH antibodies. This treatment caused a significant increase in CYTO-ID puncta in TH-positive mDAN cell bodies in comparison with the DMSO vehicle controls (Figure 6B). Lentiviral GFP-MAP1LC3B and GFP-ATG5 expression each worked effectively for live-cell imaging (Figure 6C,D), but GFP-MAP1LC3B puncta were not well retained following fixation (data not shown) precluding correlation with anti-TH staining of fixed cultures.

Immunoblotting demonstrated increased MAP1LC3B lipidation in mDAN cultures treated for 4 h with AZD8055 alone (not significant) and AZD8055 in the presence of BafA1 (significantly increased) (Figure 6E). Evidence for SQSTM1 turnover in AZD8055-treated mDAN cultures (not significant) and stabilization in the presence of BafA1 (significantly different) was also obtained in treated mDAN cultures (Figure 6E). Using EM, we observed evidence of autophagosomes in the neurites of untreated mDAN cultures, with presumed pre-synaptic vesicle clusters apparent at growth cone-neurite intersections (Figure 7A). We could also demonstrate robust increases in autolysosomal vacuole abundance in neuronal cell bodies treated with BafA1 in the absence or presence of AZD8055 (Figure 7B,C). Interestingly, and consistent with the lack of a significant change in MAP1LC3B lipidation in AZD8055-treated cultures (Figure 6E), there was no apparent quantitative change in autolysosomal cytoplasmic volume occupancy in the cell bodies of neurons treated with AZD8055 alone (Figure 7B,C). Together, these observations suggest basal and stimulated efficient autophagic flux in mDANs. Finally, to better understand the behavior of neurons in our mDAN cultures, we measured axonal growth dynamics in the absence or presence of BafA1 (to block autophagic flux). Autophagy is known to influence growth cone stability via the turnover of microtubule regulators [63]. In our cultures, axonal basal growth rates were measured to be ~0.8 μm/min, increasing marginally to ~1.0 μm/min in the presence of BafA1 (Figure 7D,E).

### Assessing mitochondrial properties and mitophagy quality control in mDANs

**Figure 8.**
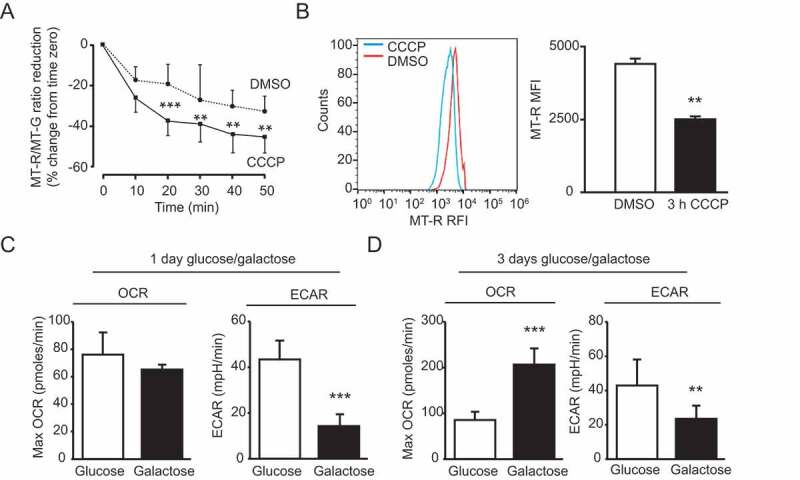
Characterization of mitochondrial properties in day 60 mDAN cultures differentiated from NAS2 hiPSCs using the optimized monolayer culture. (A) Imaging mitochondrial membrane potential collapse. Neurons were loaded with MT-R and MT-GR then imaged by wide-field fluorescence microscopy in the absence or presence of 10 μM CCCP. Data show means ± SD of 25 individual neurons from a single neuralization, verified by post-fix anti-TH labeling (not shown). (B) Measurements of mitochondrial membrane potential collapse in neuronal cultures by flow cytometry. mDAN cultures were transduced with lentiviruses expressing GFP from the SYN1 promoter, and individual GFP-expressing neurons loaded with MT-R were measured for mitochondrial membrane potential by flow cytometry. MT-R fluorescence was compared in control (DMSO) and CCCP-treated cells. (C, D) Seahorse Bioanalyzer measurements of OCR and ECAR in mDAN cultures grown for 1 d (C) and 3 d (D) in either glucose or galactose media. ** P < 0.01; *** P < 0.001

We used several tools for proof-of-principle analysis of mitochondrial properties in hiPSC-derived mDANs. Firstly, we monitored CCCP-induced mitochondrial membrane potential collapse by live-cell imaging (Figure 8A), and flow cytometry (Figure 8B). For live imaging, we loaded 60-d mDAN cultures with MT-R (membrane potential-dependent CMXRos) and MT-GR (membrane potential-independent) dyes [64], and measured fluorescence intensities in regions of interest containing individual cell bodies as a function of initial fluorescence intensity. Cells were confirmed to be mDANs by fixing and labeling with anti-TH antibodies at the end of the experiment (not shown). Figure 8A shows the decline in fluorescence (MT-R/MT-GR intensity) plotted as % reduction, confirming that 10 μM CCCP caused mitochondrial membrane potential collapse in human hiPSC-derived mDANs. We next tested whether flow cytometry could be used as a rapid, objective tool to monitor mitochondrial membrane potential in mDAN cultures. Here, population-level measurements are necessary, emphasizing the need for cultures with high numbers of verified mDANs. To ensure that we measured only neurons, we transduced cultures with lentiviruses expressing GFP from the *SYN1* promoter. Thus, we could be confident that ~70-80% of cells analyzed would be TH-positive mDANs (Figure 1D). We treated cultures for 3 h with 10 μM CCCP or vehicle control (DMSO), labeled with MT-R, then dissociated into individual cells using Accutase before being analyzed on a BD Influx cell sorter. Events were sequentially gated on forward/side scatter, Draq7 fluorescence (for viable cells), and GFP (to assess neurons only)/MT-R fluorescence (not shown). MT-R fluorescence was then quantitated, revealing that CCCP addition caused a significant drop in mitochondrial membrane potential in neurons, as expected (Figure 8B). On preparing neuronal cultures for flow cytometry, damage to neurites will inevitably occur, which may have an impact on overall cell viability/stress. It is, therefore, important to consider any data obtained using such an approach alongside orthogonal techniques.

Simultaneous measurements of oxygen consumption (OCR) and extracellular acidification (ECAR) rates provide valuable information about mitochondrial oxidative phosphorylation (OXPHOS) and aerobic glycolysis rates, respectively. We used a Seahorse XFp device to obtain baseline measurements of OXPHOS and glycolysis in 70-d mDAN cultures grown in the presence of either glucose or galactose (for 1 or 3 d) to force usage of mitochondrial OXPHOS (Figure 8C,D). FCCP concentration was optimized at 1 μM. Following 1-d growth in galactose, maximum OCR measurements were not significantly different from the glucose-treated samples, but ECAR was significantly reduced (Figure 8C). By contrast, after 3-d growth in galactose media, mDAN cultures had significantly higher maximum OCR levels (Figure 8D), suggesting that this incubation length was sufficient to trigger a switch toward OCR dependency. This analysis demonstrates the value of Seahorse measurements of hiPSC-derived mDAN cultures; further work will be needed to determine the relative influence of neurons and astrocytes in these metabolic shifts.

**Figure 9.**
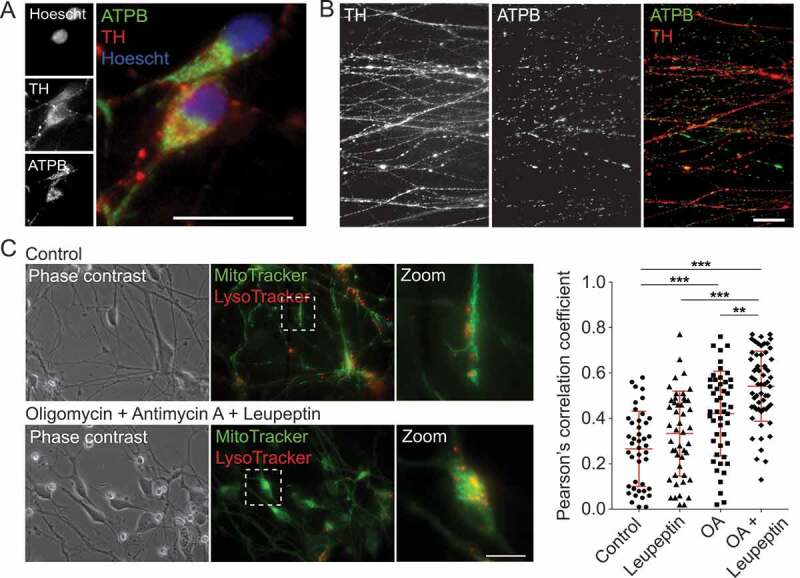
Examination of mitochondrial properties and quality control in mDANs differentiated from NAS2 hiPSCs. (A, B) Examples of neurons labeled with anti-TH and anti-ATPB (mitochondrial marker) antibodies. Cell bodies (A) and neurites (B) are shown. (C) Mitophagy analysis by colocalization of MT-G and LT-R in live mDAN cultures. To the left, example images of control and OA + Leupeptin treated neuronal fields are shown. To the right, data points show individual cells from 3 separate experiments. ** P < 0.01; *** P < 0.001. Bars: 10 μm

Analysis of baseline mitochondrial morphometric parameters in fixed, untreated TH-positive mDANs, showed that mitochondrial length ranged from 0.8 to 3.2 μm (average = 2.13 ± 0.6 μm), with mean linear axonal distance between mitochondria being 4.1 ± 2.2 μm (mean ± SD for 60 TH-positive axons; 245 mitochondria) (example images in Figure 9A,B). To measure mitochondrial dynamics by live-cell imaging, MT-R stained mDAN cultures were imaged for up to 16 h, and were subsequently fixed and stained with anti-TH antibodies for retrospective confirmation of mDAN identity. Transport rates for motile mitochondria were measured to be 0.6 ± 0.2 μm/s (50 motile mitochondria measured across 3 imaging dishes) (data not shown). To provide proof-of-principle analysis of mitophagy in living human mDAN cultures, we measured co-localization of MT-GR and LysoTracker red (LT-R) in neuronal cell bodies in mDAN cultures following 24 h treatment with oligomycin (ATP synthase [complex V] inhibitor) with antimycin A (complex III inhibitor) (OA), in the absence or presence of leupeptin (lysosomal protease inhibitor). The data showed that mitophagy events increased significantly in the presence of these inhibitors, with evidence of flux revealed in the OA + leupeptin data set (Figure 9C).

## Conclusions and future perspectives

The use of iPSCs to generate neurons for autophagy-related studies of neurodegenerative diseases including PD is widespread and has been reviewed extensively [39,65–67]. It is clear that there are substantial differences in mDAN differentiation protocols being applied, and varying standards for assessing mDAN identity/contribution in culture, highlighting a need for greater consistency. mDANs can be induced from iPSCs by overexpression of LMX1A. Using such an approach, neurite morphology defects were reported in LRRK2^G2019^ ^S^ (leucine-rich repeat kinase 2) mutant cells, corresponding with evidence of failed autophagic flux [59]. Proportions of mDANs were relatively low in this study, however (9-29%), and direct correlation with mDAN reporters was not shown for all assays [59]. The LMX1A overexpression protocol was also used to study chaperone-mediated autophagy (CMA) defects in dopaminergic neurons harboring the LRRK2^G2019^ ^S^ mutation [68]. Using an embryoid body/neurosphere protocol [69,70], Fernandes et al. generated mDANs carrying the GBA^N370^ ^S^ mutation (a PD risk factor), obtaining ~30% TH-positive/TUBB3-positive neurons [71]. They applied anti-TH staining in immunoelectron microscopy to reveal altered lysosomes morphology consistent with impaired autophagic flux in dopaminergic neurons [71]. The same differentiation protocol has been used to study mitophagy in mDANs with mutations in PRKN or PINK1 [72,73], although in one study, low numbers of TH-positive cells were reported (11-16%) and midbrain identity was not confirmed [72]. By including FACS-sorting with CXCR4/CD184^high^/CD44^−^ markers to enrich for iPSC-derived mDANs, the TH+ neuronal population has been raised to ~50%, and in this study, the mt-mKeima reporter was used to measure mitophagy in PRKN mutant cells, although correlation with mDAN markers was not shown [74]. In neurosphere iPSC-derived mDANs, the PARK7/DJ-1^G192^ ^C^ mutation was shown to influence GBA activity and lysosomal function [75]. TH-positive cells ranged from ~30% (day 30) to ~60% (day 90) of the total cell population in this study [75]. Using an WNT1-FGF8-retinoic acid patterning protocol, Hsieh et al. studied mitochondrial dynamics and mitophagy in iPSC-derived DA neurons with the LRRK2^G2019^ ^S^ mutation [76]. Mitochondrial stress parameters and attenuated OPTN and GFP-LC3 recruitment to damaged mitochondria were reported, and mt-mKeima used to demonstrate delayed mitophagy; however, mDANs contributed only 10-12% of cells in the population, meaning that only a small proportion of cells analyzed were likely to be true mDANs [76].

The monolayer-based neural differentiation protocol that we have presented here reliably and reproducibly generates ~70-80% mDANs, in a dispersed culture that is amenable to diverse imaging and functional assessments of autophagy/mitophagy-related parameters. The high proportion of confirmed mDANs achievable when using this protocol means that population-based assays are possible, since the contribution of mDANs to any changes in parameter will be substantial. The presence of astrocytes within maturing cultures likely provides support for mDANs within the culture, but this should nevertheless be considered particularly when carrying out population-based assessments and/or when stressing cultures to analyze mDAN responses since astrocytes can amplify/elicit neuronal cell stress themselves. Furthermore, the dispersed nature of the mDANs generated using this protocol means that fixed and live-cell imaging of autophagy processes can be carried out as long as care is taken during fixation and image interpretation. As a xeno- and feeder-free system, this methodology has the potential to be adapted for transplantation studies. We suggest that judicious application of this monolayer protocol for dedicated PD research and/or as part of functional validation in an important disease-relevant model system can be a very useful facet for autophagy labs.

## Materials and methods

### Reagents and antibodies

Seahorse XF base medium (Agilent Technologies, 103193–100); Seahorse XFp FluxPax (Agilent Technologies, 103022–100); Accutase (Thermo Fisher Scientific, A11105-01); ascorbic acid (Sigma, A5960); AZD8055 (Stratech Scientific, S1555); B27 (Thermo Fisher Scientific, 17504–044); bafilomycin A_1_ (BafA1; Enzo Scientific, CM110-0100); human recombinant BDNF (Preprotech, 450–02); carbonyl cyanide m-chlorophenylhydrazone (CCCP; Sigma, C2759; used at 10 μm); oligomycin (Sigma, O4876; used at 10 μM); antimycin A (Sigma, A8674; used at 1 μM); CHIR99021 (Axon Medchem, 1386); CytoID (Enzo, ENZ-51031); 1,4-diazabicylo[2.2.2]octane (DABCO; Sigma, D27802); 4ʹ,6-diamidino-2-phenylindole (DAPI; Sigma, D9542); DAPT (Tocris, 2634); db-cAMP (Sigma, D6546); DMEM-high glucose (Sigma, D5796); DMEM-F12 w/o glucose (Biowest, L0091); DMEM/F-12 + Glutamax (Thermo Fisher Scientific, 31331–028); KnockOut^TM^ DMEM (KO-DMEM) (Thermo Fisher Scientific, 10829018); KnockOut^TM^ serum replacement (KO-SR; Thermo Fisher Scientific, 10828028); Essential 8 medium/E8 supplement (Thermo Fisher Scientific, A1517001); F12 + Glutamax (Thermo Fisher Scientific, 31765); FGF2 (PeproTech, 100-18B); FGF8/FGF8A (R&D Systems, 4745-F8); FN1 (Sigma, F0162); galactose (Sigma, G0750); human recombinant GDNF (Peprotech, 450–10); Glutamax (Thermo Fisher Scientific, 35050–038); Hoechst 33342 (Thermo Fisher Scientific, 62249); human INS (Sigma; I9278); laminin (Sigma; L2020); LDN193189 (Sigma; SML0559); LysoTracker Red (LT-R; Thermo Fisher Scientific, L7528); mitomycin C (Tocris, 5238); MitoTracker Green (MT-GR; Thermo Fisher Scientific, M7514); MitoTracker Red (MT-R; CMXROS; Thermo Fisher Scientific, M7512); monothioglycerol (Sigma, 35,050); N2 (Thermo Fisher Scientific, 17502–048); neurobasal (Thermo Fisher Scientific, 21103–049); non-essential amino acids (NEAA; Thermo Fisher Scientific, 11140–035); PD0325901 (Axon, 1408); penicillin/streptomycin (Sigma, P4333); poly-L-ornithine (PO; Sigma, P4957); polyhema (Sigma, P3932); purmorphamine (Calbiochem, 540220); RevitaCell (Thermo Fisher Scientific, A2644501); SB431542 (Tocris, 1614); human recombinant SHH (sonic hedgehog; SHH-C24II; R & D Systems, 1845SH025); SYBR Green (Thermo Fisher Scientific, 4310179); TGFB3 (Peprotech, 100-36E); TF/transferrin (Sigma, T8158); VTN/vitronectin (Thermo Fisher Scientific, A14700); Y27632 (Tocris, 1254).

Antibodies used were: rabbit anti-TH (Millipore, AB152); mouse anti-TH (Santa Cruz Biotechnology, sc-25269); anti-TUBB3 (TUJ1) (Bio-legend, 801201); anti-FOXA2 (Santa Cruz Biotechnology, sc-101060); anti-LMX1A (Millipore, AB10533); anti-LMX1B (Proteintech, 18278); anti-TBR1 (Abcam, AB31940); anti-WIPI2 (BioRad, MCA5780 GA); anti-MAP1LC3B (Sigma, L7543); anti-SQSTM1 (Abnova, H00008878); anti-ATP5F1B (Abcam, AB14730); anti-DLG4 (Neuromab, 75028); anti-S100B (Abcam, AB868); anti-SYP (Abcam, AB32127); anti-SLC17A7 (Synaptic Systems, 135303); Alexa Fluor 488 anti-rabbit (Thermo Fisher Scientific, A21206); Alexa Fluor 568 anti-rabbit (Thermo Fisher Scientific, A10042); Alexa Fluor 488 anti-mouse (Thermo Fisher Scientific, A21202); Alexa Fluor 568 anti-mouse (Thermo Fisher Scientific, A10037); Alexa Fluor 647 anti-rabbit (Thermo Fisher Scientific, A31573); Alexa Fluor 647 anti-mouse (Thermo Fisher Scientific, A31571); anti-rabbit HRP (Jackson Immunoresearch, 111035144); anti-mouse HRP (Jackson Immunoresearch, 115035003).

### Cell-lines and culture conditions

All of the functional data presented in this study was obtained using the “Normal SNCA” (NAS2) hiPSC line [77] (a gift from Dr. Tilo Kunath, Center for Regenerative Medicine, University of Edinburgh; used at passages 40–70). We used several additional stem cell-lines during this development of our system, including AST23 SNCA triplication 23 hiPSC line originating from a member of the same family from which the NAS2 was derived (from Dr. Tilo Kunath [77]); Sheffield 6 (SHEF6) ES line [78] (acquired from the UK-Stem Cell Bank; used between passage 45–53); Michigan State University Human 001 (MSU-H001) hiPSC line [79] (a gift from Prof. Jose Bernardo Cibelli, Cellular Reprogramming Laboratory, Michigan State University; used between passages: 51–70); Mouse embryo fibroblasts (MEFs) were derived from pregnant mice sacrificed at gestation day 12–13 (E12-E13). These were inactivated by a 2 h incubation (37°C/5% CO_2_) in mitomycin c diluted in MEF media to a final concentration of 10 μg/ml.

For feeder-dependent human stem cell culture, stem cells were maintained on MEF feeder layers in stem cell media consisting of KO-DMEM supplemented with 20% KO-SR, 1% Glutamax, 1% Pen/Strep, 0.2% β-mercaptoethanol (Sigma, M6250), 1% NEAA and 20 ng/ml FGF2. Cells were allowed to form colonies and expand to up to 80% confluency (for approximately 8 to 10 d). Media was changed daily. Colonies were pruned every 4 d from differentiated cells and from the surrounding MEFs. For feeder-free culture, ESCs and hiPSCs were plated on to VTN-coated plates containing Essential 8 (E8) xeno/feeder-free medium, at a density of 4–5 × 10^4^ cells/cm^2^. For coating, 5 μg/ml VTN (diluted in PBS; Thermo Fisher Scientific, 14190–144) was applied to 60 mm diameter tissue culture dishes for 1 h at room temperature. Culture medium (E8) was changed daily until cells reached ~80% confluency (usually 4 d after plating). The cells were then passaged, frozen, or differentiated. A detailed protocol for feeder-free hiPSC maintenance can be found in [80].

### *The optimized monolayer differentiation protocol for human mDANs (see* *Figure 1A*)

hiPSCs were cultured in E8 medium for up to 4 d, before transferring to neural induction medium, comprising N2B27 medium supplemented with 100 nM LDN193189, 10 μM SB431542, 200–500 ng/ml SHH and 0.6–1.0 μM CHIR (see Results/Discussion); this was considered neuralization day 0. Cells were passaged on PO/laminin-coated plates at neuralization days 3 and 7 using Accutase, at a density of 4 × 10^4^ cells/cm^2^ in neural induction medium supplemented with 10 μM Y27632 or 1:100 RevitaCell [80]. Media was changed daily and was replaced with progenitor expansion medium (N2B27 supplemented with 20 ng/ml BDNF, 20 ng/ml GDNF, 0.2 mM AA and 10 μM Y27632) on neuralization day 11. When a full monolayer of cells was established, cells were passaged on PO/laminin-coated plates in progenitor expansion medium (cells can be expanded and passaged weekly in this manner beyond day 50, with certain considerations [80]). From day 16 onwards, terminal differentiation was initiated by passaging and plating cells at a density of 5,000 cells/cm^2^ in 10–50 μl droplets on PO/laminin-coated coverslips in terminal differentiation medium, comprising N2B27 supplemented with 20 ng/ml BDNF, 20 ng/ml GDNF, 0.2 mM ascorbic acid, 500 μM db-cAMP, 10 μM DAPT and 10 μM Y27632. Cells were allowed to attach for ~1 h before coverslip-containing wells were carefully flooded with the same media. Neurons were fed at 3-d intervals by exchanging with ~50% N2B27 media supplemented with 20 ng/ml BDNF, 20 ng/ml GDNF, 0.2 mM ascorbic acid, 500 μM db-cAMP and 10 μM DAPT. A detailed, stepwise differentiation protocol is available [80].

#### Light microscopy

Droplets of cells were plated on PO/laminin pre-coated coverslips in 4-well plates (Nunclon Delta Treated 4-Well IVF Dish; Thermo Fisher Scientific, 144,444) or live imaging dishes (35 mm glass-bottomed dishes; MatTek, P35 G-0.170-14-C) and allowed to terminally differentiate for 7–14 d. For fixed imaging, differentiated neurons were washed with PBS (Sigma, D8537), then fixed in 4% paraformaldehyde for 20 min at room temperature, or in 100% −20°C methanol for 5 min. After fixation, cells were washed twice with PBS and permeabilized (paraformaldehyde-fixed cells only) using 0.2% Triton X-100 (Sigma, T9284), 5% normal donkey serum (Sigma, S30-M), and 2% bovine serum albumin (Sigma, A9647) in PBS for 1 h at room temperature. Cells were incubated with primary antibody either overnight at 4°C, or for 1 h at room temperature, according to the manufacturer’s recommendations (see [80]). Following further PBS washes, cells were incubated with fluorescent secondary antibodies in the dark for 1 h at room temperature. Nuclei were stained with 10 μg/ml Hoechst 33,342 or 100 ng/ml DAPI. Coverslips were mounted on glass slides using Mowiol supplemented with 25 mg/ml DABCO anti-fade. For live-cell imaging, cells were plated on PO/laminin pre-coated and allowed to terminally differentiate for 10 d before imaging on the Olympus IX-51 platform. In order to confirm the presence of DANs, “on stage” immunolabeling was performed with stage positions recorded using MetaMorph. For mitochondrial and lysosomal labeling, MT-GR, MT-R and LT-R dyes were applied: before labeling, the cells were washed once with PBS, followed by 30 min incubation (37°C/5% CO_2_) with 50 nM dyes in N2B27 supplemented with terminal differentiation and maturation factors. Next, the cells were washed twice with PBS, which was replaced with cell culture media. For CYTO-ID staining, cells were incubated for 30 min in N2B27 containing 2 μl/ml of CYTO-ID Green. Cells were then washed and fixed with 4% paraformaldehyde and processed for immunofluorescence imaging as above.

Wide-field microscopes used: Leica DMRD upright fluorescence microscope fitted with a Leica DFC450 C camera 5 Megapixel CCD sensor, with image capture and analysis performed using a Leica Application Suite software; Olympus IX-71 inverted microscope hosting a 60x Uplan Fluorite objective (0.65–1.25 NA, oil) and fitted with a CoolSNAP HQ CCD camera (Photometrics), with image capture and analysis performed using MetaMorph software (Molecular Devices). Confocal microscopy was performed using a Leica SP5-AOBS laser-scanning confocal microscope (63x oil immersion objective, 1.4 NA; or 100x oil immersion objective, 1.4 NA).

### Electron microscopy

For electron microscopy, mDANs were seeded on 35 mm glass-bottomed dishes as for light microscopy (above), and treated with AZD8055 and/or BafA1 for 4 h. Cells were fixed by the addition of 2% glutaraldehyde (EM grade) for 15 min at room temperature. Following 3x washes with 0.1 M Na Cacodylate buffer, cells were osmicated (1% OsO_4_ with 1.5% K_3_[Fe{CN}_6_] in 0.1 M Na Cacodylate), washed, then dehydrated in an ethanol series before being embedded in Epon resin. Hardened resin was removed from the imaging dish, blocks were trimmed, and ~70 nm ultrathin sections cut using an Ultracut S ultramicrotome (Leica). Sections were post-stained in uranyl acetate and lead citrate and viewed using a Technai 12 120 kV transmission electron microscope (FEI). Images were obtained using a Ceta 4 k x 4 k CCD camera and processed using Fuji software.

### Lentiviruses

Lentiviruses were produced in HEK293 T as described [81]: (i) PXLG3 GFP-LC3 (Lane lab); (ii) PRRL human SYN1-GFP (gift from Prof. James Uney [University of Bristol]); (iii) PLVX EF1A GFP-ATG5 (Lane Lab). 45-50-d-old neural progenitors were transduced with lentiviruses 2–4 d after plating. After 3 d, media was replaced with fresh N2B27 media supplemented with BDNF, GDNF, AA, db-cAMP and DAPT for a further 3 d. Following a final media change, infected cells were imaged live or used for flow cytometry.

### Quantitative real-time polymerase chain reaction (qRT-PCR)

Cells were plated in triplicate on 12-well plates at a density of 4x10^4^/cm^2^. RNA was extracted by using the QIAGEN RNeasy kit following the manufacturer’s instructions. Eluted RNA was measured by a Nanodrop spectrophotometer (Thermo Fisher Scientific). cDNA was prepared using a High Capacity RNA-to-cDNA Kit (Thermo Fisher Scientific, 4,387,406) according to the manufacturer’s instructions. cDNAs were amplified using the SYBR Green Master Mix (Thermo Fisher Scientific, 4,309,155) and specific amplification primers (250 nM final concentration) according to the manufacturer’s protocol, using a StepOnePlus Real-Time PCR device (Applied Biosystems). For 10 μl reactions, 2 μl of cDNA was mixed with 2 μl of RNase-free H_2_O, 5 μl of SYBR Green, 0.5 μl forward and 0.5 μl reverse primers. Cycle conditions set as follows: an initial denaturation at 95°C for 10 min followed 40 cycles with 95°C for 15 s (denaturation), 60°C for 30 s (annealing) and 60°C for 30 s (elongation). The relative quantification of gene expression was performed via automatic calculation of the cycle threshold (Ct) values for each target gene and was normalized for the expression of the housekeeping gene *GAPDH* (glyceraldehyde-3-phosphate dehydrogenase), and was calculated using the 2^(-ΔΔCt)^ method [82].

The following primers were used (Gene: 5ʹ-3ʹ forward/5ʹ-3ʹ reverse): *DDC*: ggggaccacaacatgctgctcc/aatgcactgcctgcgtaggctg; *ATG16L1*: cagttacgtggcggcaggct/acaacgtgcgagccagaggg; *ATG5*: agcaactctggatgggattg/cactgcagaggtgtttccaa; *CORIN*: catatctccatcgcctcagttg/ggcaggagtccatgactgt; *FOXA2*: ctgggagcggtgaagatgga/acgtacgacgacatgttcatggag; *GAPDH*: ttgaggtcaatgaaggggtc/gaaggtgaaggtcggagtca; *KCNJ6* (*GIRK2*): atggatcaggacgtcgaaag/atctgtgatgacccggtagc; *LAMP2*: cgttctggtctgcctagtcc/cagtgccatggtctgaaatg; *LMX1A*: agagctcgcctaccaggtc/gaaggaggccgaggtgtc; *NANOG*: ttgggactggtggaagaatc/gatttgtgggcctgaagaaa; *NR4A2*: gtgttcaggcgcagtatgg/tggcagtaatttcagtgttgg; *POU5F1* (*OCT4*): gacaacaatgagaaccttcaggaga/ctggcgccggttacagaacca; *OPTN*: tgctgagtccgcacataga/gggtccatttcctgtgctt; *SQSTM1*: ctgggactgagaaggctcac/gcagctgatggtttggaaat; *PINK1*: gcctcatcgaggaaaacagg/gtctcgtgtccaacgggtc; *TH*: gccgtgctaaacctgctctt/gtctcaaacaccttcacagctc.

### Flow cytometry

50-55-d-old differentiated neuronal progenitors were plated in 12-well plates at a density of 6 × 10^4^ cells/cm^2^ and allowed to terminally differentiate for 10 d. 3 d after plating, cells were transduced with PRRL human SYN1-GFP lentiviruses. 10 d post-plating, cells were treated for 3 h with DMSO (vehicle control) or 10 μM CCCP and were then washed once with PBS and probed with 50 nM MT-R for 30 min (37°C/5% CO_2_). Following 2 washes with PBS, MT-R labeled cells were dislodged into single cells by 10 min incubation in Accutase (37°C/5%CO_2_), centrifuged (100 x g for 1 min) and resuspended in fresh pre-warmed DMEM. Flow cytometry was performed using a BD Influx cell sorter (BD Biosciences). Briefly, over 50,000 viable cells were recorded using the DRAQ7 DROP & GO viability dye (Invitrogen, D15107). Cells were excited using 488 nm, 552 nm and 640 nm lasers, and fluorescence emissions were captured for GFP (488 nm 530/40 nm BP), MT-R (552 nm 610/20 nm BP) and DRAQ7 (640 nm 750 LP) respectively. Data were analyzed using FlowJo v10.3 software (Treestar).

### Seahorse XFp bioanalyzer

To measure oxygen consumption rates (OCR) and extracellular acidification rates (ECAR) in the presence of glucose or galactose, an Agilent Seahorse XFp Analyzer (Santa Clara, USA) with cell energy phenotype test kit was used (according to the manufacturer’s instructions). 54-d-old neural progenitors were plated on 8-well Seahorse XFp Cell culture mini-plates (Agilent, 103022–100) at a density of 4 × 10^4^ cells/cm^2^ and allowed to differentiate in N2B27 media supplemented with BDNF, GDNF, AA, db-cAMP and DAPT. Depending on the experimental conditions, 1 or 3 d prior to the assay, media was replaced with Seahorse bioenergetics media consisting of 1:1 DMEM-F12 (no glucose) and Neurobasal (no glucose) supplemented with 20 ng/ml BDNF, 20 ng/ml GDNF, 200 μM AA, 500 μM db-cAMP, 10 μM DAPT, 0.25 mM sodium pyruvate and 10 mM galactose or 10 mM glucose (instead of galactose). On the day of the assay, culture media was replaced with Seahorse XF base medium supplemented with 1 mM sodium pyruvate, 2 mM glutamine, and 10 mM glucose or 10 mM galactose (depending on the condition; pH 7.4) and the cells were incubated at 37°C (without CO_2_) for 1 h before the assay. Data was extracted and analyzed automatically by the Seahorse Wave software.

### Statistical analysis

Mean differences were analyzed by student’s t-test, one-way and two-way ANOVA (analysis of variance) followed by Tukey’s (all values compared with each other) or Danette’s (all values compared with a control value) multiple comparison posthoc tests. Statistical significance was determined as *p < 0.05, **p < 0.01 and ***p < 0.001 using GraphPad Prism 6 software (Graph Pad Software Inc., CA).

## Supplementary Material

Supplemental Material
