# Supplementary figures and images for: A monolayer hiPSC culture system for autophagy/mitophagy studies in human dopaminergic neurons
Source: Autophagy. 2020 Apr 14;17(4):855–71. doi: 10.1080/15548627.2020.1739441 (PMC8078667; doi:10.1080/15548627.2020.1739441)

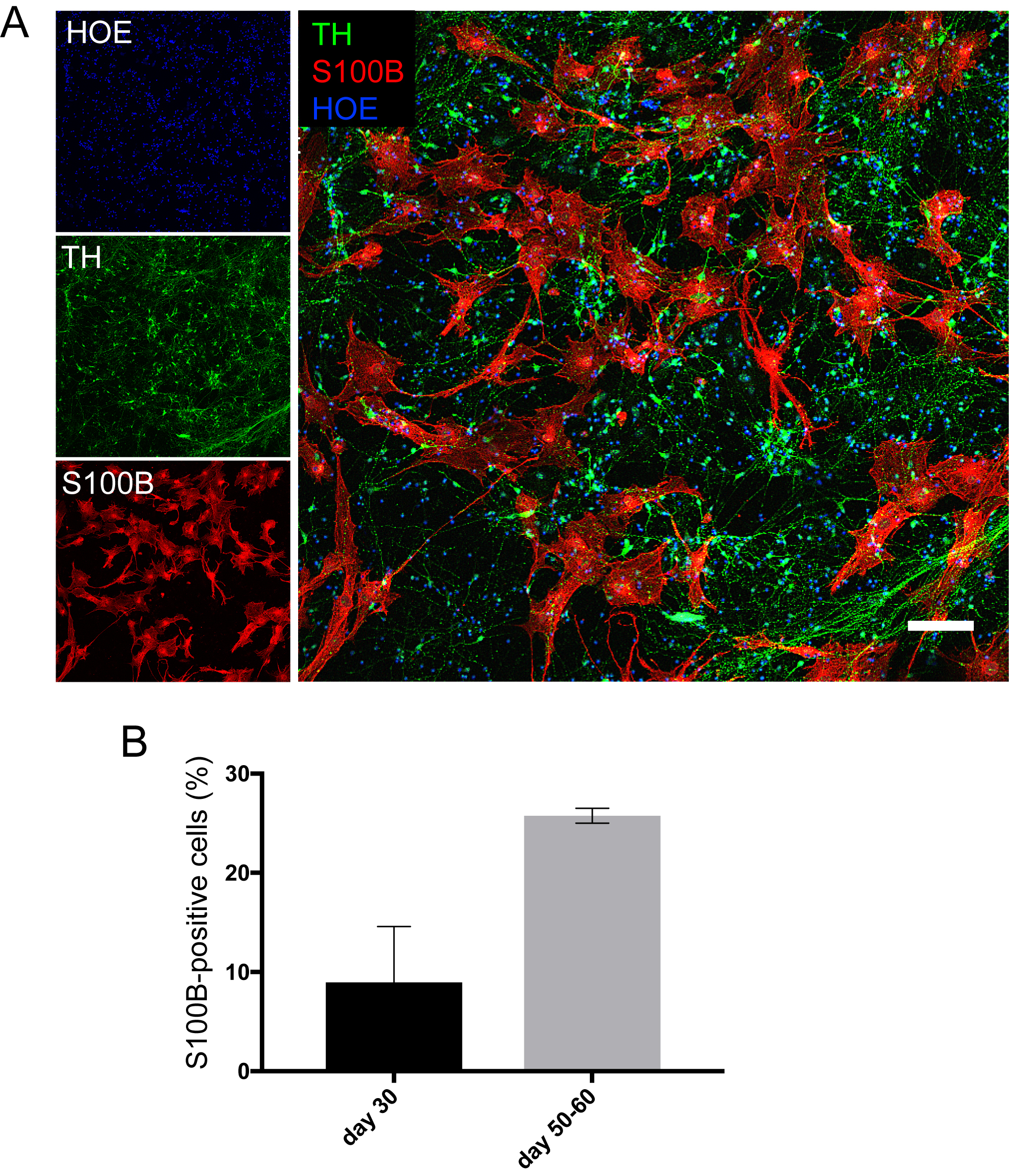

Supplement: Supplemental Material [file KAUP_A_1739441_SM4705.zip › Suppl Fig 5new.jpg]
